# Temporal dynamics of bacterial microbiota in the human oral cavity determined using an *in situ* model of dental biofilms

**DOI:** 10.1038/npjbiofilms.2016.18

**Published:** 2016-08-10

**Authors:** Nanako Wake, Yoko Asahi, Yuichiro Noiri, Mikako Hayashi, Daisuke Motooka, Shota Nakamura, Kazuyoshi Gotoh, Jiro Miura, Hiroyuki Machi, Tetsuya Iida, Shigeyuki Ebisu

**Affiliations:** 1 Department of Restorative Dentistry and Endodontology, Osaka University Graduate School of Dentistry, Osaka, Japan; 2 Division of Cariology, Operative Dentistry and Endodontics, Department of Oral Health Science, Niigata University Graduate School of Medical and Dental Sciences, Niigata, Japan; 3 Research Institute for Microbial Diseases, Osaka University, Osaka, Japan; 4 Department of Bacteriology, Okayama University Graduate School of Medicine, Dentistry and Pharmaceutical Sciences, Okayama, Japan; 5 Division for Interdisciplinary Dentistry, Osaka University Dental Hospital, Osaka, Japan; 6 Osaka University Dental Technology Institute, Osaka, Japan

## Abstract

Numerous studies on oral biofilms have been performed *in vitro*, although it is difficult to mimic the oral environment. Here we used an *in situ* model to conduct a quantitative analysis and comprehensive identification of bacterial communities over time by performing deep sequencing of 16S rRNA genes. We show here that the number of viable bacteria in supragingival biofilms increased in two steps. Using scanning and transmission electron microscopy, as well as confocal laser scanning microscopy, we detected gram-positive cocci during the first 8 h. The biofilm was subsequently covered with a thick matrix-like structure composed of different bacterial morphotypes that diversified as the number of bacteria increased. *Streptococcus* accounted for >20% of the population until 16 h, and obligate anaerobes such as *Fusobacterium, Prevotella* and *Porphyromonas* predominated after 48 h, and this increase was statistically significant after 96 h (*P*<0.05). Together, our data demonstrate that an initial population of facultative anaerobic bacteria was replaced with a population of gram-negative anaerobic bacteria during oral biofilm formation. This study, therefore, contributes to a comprehensive understanding of the composition of the bacterial microbiota involved in the health of the human oral cavity.

## Introduction

Biofilms are complex structures cooperatively formed by bacterial communities comprising multiple species that grow on a solid surface.^1^ The oral cavity is inhabited by ⩾700 species of bacteria^2,3^ that form dental biofilms in various places such as the tooth surface and gingival sulcus. Biofilm formation is affected by diverse intraoral environmental and host factors such as host immunity, pH, enzymes, saliva and antibiotics.^4^ Biofilms are a major cause of caries and periodontal disease, and the interaction of biofilm microbial populations affects bacterial virulence.^5^ Therefore, it is important to understand the mechanism of biofilm formation to prevent and treat dental diseases. Previous studies of dental biofilms investigated the mechanism of biofilm formation, gene expression and exclusion-suppression methods using *in vitro* models that used one or a few bacterial species.^6–10^ However, dental biofilms are difficult to model *in vitro* because of their species diversity and the complex environment of the oral cavity. Therefore, *in situ* models are required to understand the mechanism of human dental biofilm formation and how to mitigate their contribution to disease.

Previous analyses utilized *in situ* models of biofilm formation.^11,12^ However, these studies were primarily qualitative, relying on microscopic observations of biofilm formation, and the data are insufficient to acquire a detailed understanding of the mechanism of dental biofilm formation. Further, previous studies found that dental biofilm formation increases bacterial diversity and alters the predominant bacterial species present over time, which changes the environment in which oral disease develops.^6,13^ To understand the causes of oral diseases, the dynamics of bacterial communities in biofilms must be investigated using quantitative and metagenomic analyses. *In situ* models of biofilms offer a unique opportunity to perform these investigations.

Recently, much effort has been invested in understanding the communities of bacteria that inhabit the interior and surface of the human body. The 2012 Human Microbiome Project conducted a large-scale metagenomic analysis of the microbiota of the airway, skin, oral cavity, gut stool and vagina of healthy adults.^14^ This study revealed the characteristics of the normal microbial populations of healthy individuals for the first time and clarified the role of variations in the normal microbiota in human health and disease.

The Human Microbiome Project investigated the composition of the bacterial microbiota on the tongue surface, buccal mucosa, saliva, supragingival plaque and subgingival plaque.^15^ Next-generation sequencing was used to compare the oral microbiota of people with or without caries,^16^ as well as the subgingival bacterial microbiota of periodontal tissue of normal subjects and with subjects with gingivitis or periodontitis;^17^ however, these analyses were conducted at a single time point. Therefore, insufficient data are available to understand the dynamics of bacterial microbiota over time. Similarly, studies that used *in situ* models and 16S rRNA gene sequencing comprehensively identified bacterial species that reside in biofilms;^18,19^ however, we are unaware of any detailed investigations that addresses the changes in bacterial species over time during biofilm maturation.

To address this gap in our knowledge, here we developed an *in situ* biofilm model and used it to analyse biofilms to evaluate the characteristics of human dental biofilms over time. We used our *in situ* model of biofilms to conduct a quantitative next-generation sequencing analysis of the microbiota of experimental biofilms and comprehensively identified changes in bacterial communities over time.

## Results

### The population of biofilm-forming bacteria and the thickness of biofilms increase in two steps

**Figure 1:**
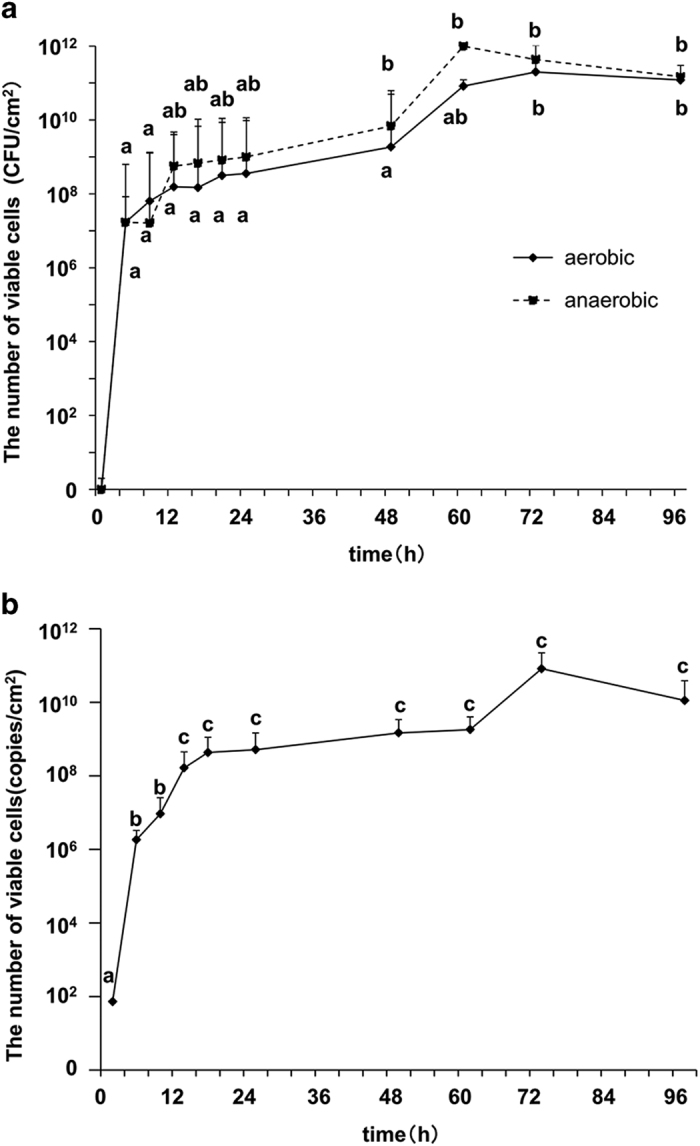
Quantification of biofilm-forming cells. (**a**) Viable bacterial cell count per unit area. Solid and dashed lines indicate bacteria incubated under aerobic or anaerobic conditions, respectively. The letters a and b represent a significant difference between the opposing letters (*P*<0.05). Significant differences in the numbers of bacteria incubated under aerobic or anaerobic conditions are shown below the solid line and above dotted line, respectively. (**b**) Real-time PCR analysis of biofilm-forming cells. The letters a, b and c represent significant difference between the opposing letters (one-way analysis of variance, Tukey–Kramer test, *P*<0.05). Data are presented as the mean±s.e.m. (*n*=10).

Viable cell counts under aerobic conditions increased rapidly during the first 12 h and increased gradually thereafter. After a statistically significant increase between 48 and 72 h, the population of viable cells plateaued (Figure 1a). Thus, the number of viable biofilm-forming cells increased in two steps. We used real-time PCR to simultaneously determine the number of bacteria present at each time (Figure 1b). First, the number of bacteria increased significantly from 1 to 12 h (*P*=0.0112) and increased gradually thereafter. The total number of bacteria increased again after 72 h and then plateaued. These results indicate that the increase in the total number of bacteria in a dental biofilm was similar to that of viable cells shown in Figure 1a.

**Figure 2:**
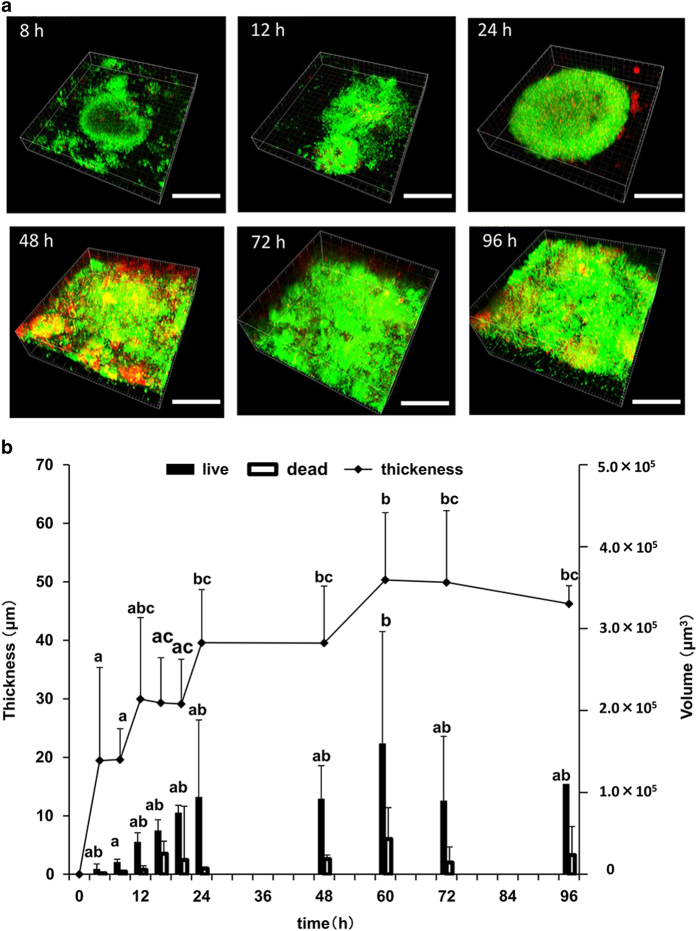
CLSM analysis of biofilms. (**a**) CLSM observations. Scale bar, 50 μm. (**b**) Determination of the thickness and volume of live and dead cells using Imaris imaging software. Black and white bars represent the volumes of live and dead cells, respectively. The letters a, b and c represent significant difference as indicated by the opposing letters (one-way analysis of variance, Tukey–Kramer test, *P*<0.05). There were no significant differences at any time between the volumes of dead cells. CLSM, confocal laser scanning microscope.

After 8 h, biofilms were observed using confocal laser scanning microscope. Biofilm thickness increased after 24 h. At 48 h, the observed biofilm-forming area was expanded, and dead cells were frequently observed in the lower layers nearest the hydroxyapatite (HA) disk, and the number of live cells was increased in the upper layers (Figure 2a). Biofilm thickness increased and attained its maximum thickness (50 μm) at this time. Biofilm thickness decreased slightly at 72 h (Figure 2b). Thus, the changes in biofilm thickness were similar to those of the viable cell count.

The volumes of live and dead cells changed similarly compared with the number of viable cells (Figure 2b). The volume of live cells increased rapidly during the first 24 h and then increased gradually until 72 h when the volumes of live and dead cells decreased slightly.

### Adherent biofilms formed on the disks

**Figure 3:**
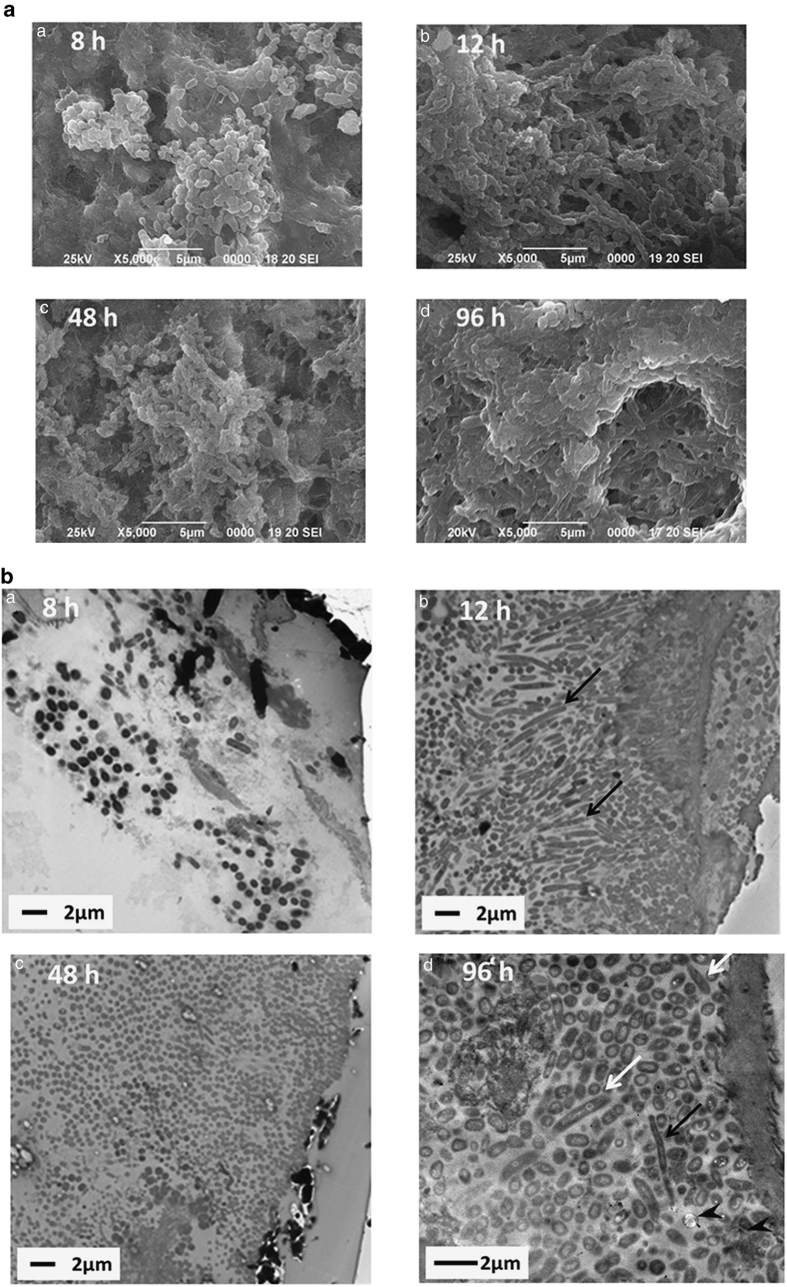
SEM and TEM observation of biofilms (**a**) SEM. (**b**) TEM. The side of the hydroxyapatite (HA) disk is shown on the right side of each image. Black arrow, filamentous bacteria; white arrow, fusiform bacteria; arrowhead, bacterial ghosts. SEM, scanning electron microscope; TEM, transmission electron microscope.

The scanning electron microscope (SEM) and transmission electron microscope (TEM) observations performed after 8 h revealed the presence of biofilms comprising gram-positive cocci (Figures 3a and b), and filamentous bacteria appeared after 12 h (Figure 3b). Subsequently, a thick matrix-like structure covered the biofilm (Figure 3a), which contained different morphotypes such as spherical, fusiform (Figure 3b) and filamentous (Figure 3b) bacteria. Moreover, dead cells called bacterial ghosts were frequently observed on the side of the HA disk (Figure 3b).

### Oral bacterial diversity changes with time in healthy individuals

**Figure 4:**
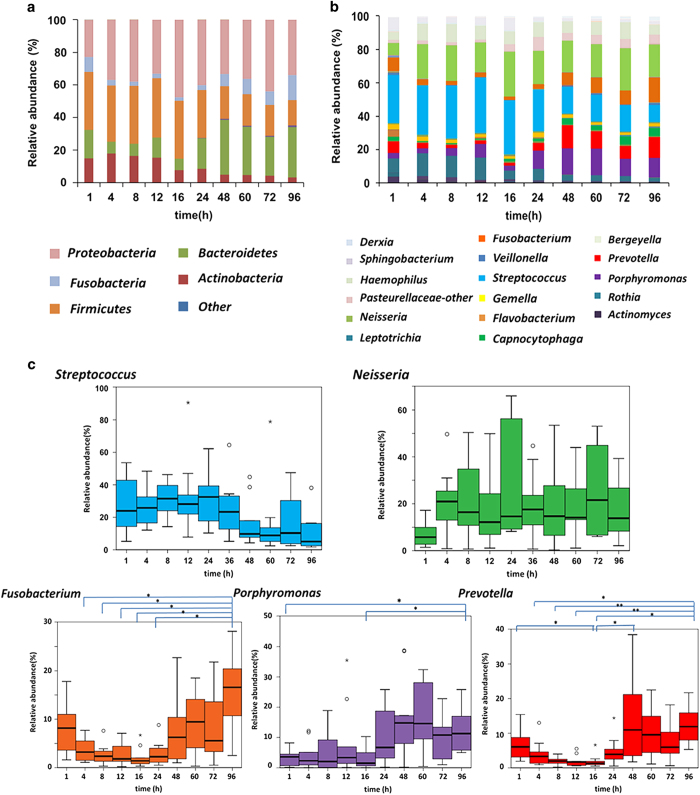
Relative abundances of bacterial taxa among the subjects. (**a**) Relative abundance of bacterial phyla in all subjects (*n*=10). This graph represents the average of data collected from all the subjects at each time point. (**b**) Proportional abundance of genera in all the subjects (*n*=10). The temporal changes in the relative amounts of each genus are indicated. The most frequently detected taxa (>1% relative abundance) in each level are shown. (**c**) Proportional abundance of five genera. Boxes extend from the 25th to 75th percentiles. Circles represent outliers. Asterisks above the whisker indicate a statistically significant difference at each time. *P<0.05; **P<0.01 (Kruskal–Wallis, Steel–Dwass test).

We next determined the change in bacterial biofilm populations over time. Similarities in bacterial populations were detected among the 10 subjects (Supplementary Figure S1). For example, the phylum *Firmicutes* was dominant initially and *Fusobacteria* and *Bacteroidetes* increased after 48 h (Figure 4a). The phylum *Proteobacteria* was the most abundant except at 1 h. *Firmicutes* accounted for >30% of the bacterial population. After 48 h, the relative abundance of *Bacteroidetes* and *Fusobacteria* increased, whereas that of *Firmicutes* and *Actinobacteria* decreased. Compared with their numbers at 1 h, the increase of *Bacteroidetes* and *Fusobacterium* and the decrease of *Firmicutes* and *Actinobacteria* at 96 h were significantly different (*P*<0.05).

*Firmicutes* was represented almost entirely by *Streptococcus*; *Fusobacteria* was almost entirely composed of *Fusobacterium* and *Bacteroidetes* was represented by *Capnocytophaga*, *Prevotella* and *Porphyromonas* (Figure 4b). Therefore, our further analyses focused on temporal changes of the most frequently detected taxa, which averaged >1% of the population (Supplementary Table S2). Together, aerobic and facultative anaerobic bacteria represented approximately 70% of recovered taxa until 24 h. After 48 h, the proportion of these taxa was reduced to 50–60%. Conversely, anaerobic bacteria accounted for ⩽20% of the taxa until 24 h and increased to 30–40% after 48 h.

To determine whether these changes were statistically significant, we restricted our analysis to the five most abundant genera that represented ⩾5% of the taxa (Figure 4c). In the initial phase of biofilm formation, *Streptococcus* accounted for ⩾20% of the taxa. After 48 h, the relative abundance of obligate anaerobes such as *Fusobacterium*, *Porphyromonas* and *Prevotella* increased significantly, and the difference was statistically significant after 96 h (*P*<0.05; Figure 4c). The population of *Streptococcus* was ⩾20% until 16 h, decreased starting at 24 h and was <5% after 96 h. In contrast, *Neisseria* accounted for >20% after 4 h, remaining constant thereafter. *Fusobacterium* began increasing in abundance after 48 h, and was significantly increased at 96 h compared with 4 h (*P*=0.0380), 8 h (*P*=0.0233), 12 h (*P*=0.0207), 16 h (*P*=0.0122) and 24 h (*P*=0.0340). The proportion of *Porphyromonas* began to increase after 48 h and was significantly higher at 96 h compared with 1 h (*P*=0.0265) and 16 h (*P*=0.0480). Moreover, the proportion of *Prevotella* began to increase after 48 h and was significantly higher at 96 h compared with 4 h (*P*=0.0482), 8 h (*P*=0.0070), 12 h (*P*=0.0092) and 16 h (*P*=0.0122).

## Discussion

The recent rapid development of techniques to analyse microbiomes has made possible major advances in the characterization of many human microbiomes, including the oral microbiota.^14,15^ However, *in situ* analyses of the population of biofilm-producing bacteria in the oral cavity only focus on the initial^18^ or later phase.^19^ Therefore, we used an *in situ* model of biofilm formation to investigate the temporal dynamics of the oral microbiome of healthy individuals. To our knowledge, the present study reveals for the first time that the number of viable bacteria in a supragingival biofilm increased in two steps. For example, the populations of certain gram-negative anaerobic bacteria rapidly increased and predominated after 48 h, which is consistent with sequencing data. Moreover, quantitative PCR analyses confirmed the biphasic increase in bacterial numbers (Figure 1). These findings are consistent with a previous quantitative analysis of biofilm formation on HA disks, which found that the number of bacteria deposited on a biofilm reaches a plateau after 4 days.^19^

Further, the thickness and volume of biofilms revealed that these temporal changes correlated with temporal changes in the number of viable bacteria (Figure 2b). However, during one period of biofilm formation, thickness did not increase with an increase in viable cell counts, which we determined was caused by the increase in the surface area of biofilms. These results suggest that dental biofilms first increase in thickness and then in area, which is followed by a further increase in thickness. After 60 h, the thickness and volume of live and dead bacteria decreased, although the biofilm covered the entire area of the HA disks. It is therefore likely that dental biofilms peeled off during this period. This may be explained by reaching a thickness limit determined by a biofilm’s three-dimensional structure. For example, the dead and viable bacterial cells were observed in the lower and upper layers, respectively, after 48 h. This finding is consistent with previous studies^20^ and indicates the importance of dead cells and debris in the initial stages of biofilm development.

Here, we sampled during three consecutive time intervals. One report that used nucleotide sequence analysis shows that in initial dental biofilms, there is no significant difference in the percentage of bacteria constituting the biofilm sampled at different day.^18^ In contrast, we are not aware of any studies that focus on mature biofilms. In the present study, when we collected samples at 12 h during the primary and secondary periods and at 48 h during the secondary and third periods, we found that there was no significant difference between the bacterial compositions of each (data are not shown). Therefore, we concluded that bias was not introduced when samples were acquired at different intervals within each of the three periods.

To determine whether our *in situ* approach accurately modelled biofilm formation *in vivo*, we performed SEM and TEM analyses (Figure 3). The artificial biofilms were morphologically similar to those that adhere to natural tooth surfaces.^21,22^ Further, the bacterial morphologies observed using TEM, which correlated with our sequencing data, demonstrate that until 12 h, the biofilms were primarily populated by gram-positive cocci, and after 48 h, we observed diverse bacterial morphologies.

Previous studies demonstrate that maturation of dental plaques correlates with increased bacterial diversity over time.^23^ However, we show here that bacterial diversity decreased during the first 16 h, subsequently increased, and became most diverse after 96 h (Supplementary Figure S2). It is considered that because the partial pressure of oxygen in biofilms decreases as they mature, the proportion of aerobic bacteria is reduced until 16 h, and only aerotolerant bacteria such as *Streptococcus* remained. Diversity increased thereafter when the biofilm became anaerobic, and anaerobic bacteria were more likely to adhere. It is possible that the accumulation of metabolites produced by aerotolerant bacteria such as *Streptococcus*, or an insufficient nutrient supply, decreased bacterial diversity during the initial phase of biofilm formation. Further investigations of environmental factors such as oxygen pressure, nutrients and pH are required to understand the changes in the bacterial diversity of biofilms.

Our sequence analysis of bacterial taxa identified variations between healthy subjects, which is consistent with previous reports.^24,25^ However, when we performed statistical analysis, certain trends were observed (Figure 4c). For example, the prevalence of *Streptococcus* during the initial phase of biofilm formation was consistent with previous investigations of initial communities, which used an *in situ* model.^18,24^ The phenotypes of streptococcal species, which enable them to become the pioneer species during biofilm formation, include their efficient attachment to the pellicle of enamel,^26,27^ as well as their ability to directly metabolize components of saliva as a nutrient source.^28^ Thus, it is likely that *Streptococcus* preferentially adhered to disks fabricated from hydroxyapatite, which is the main component of enamel.

Further Diaz *et al.*^18^ demonstrated that during the first 8 h of biofilm formation, *Streptococcus*, *Gemella* and *Neisseria* were commonly detected as the group of initial bacteria in three subjects. We show here that the genera *Streptococcus* and *Neisseria* were dominant in the early phase and that 48 h after the initiation of biofilm formation, gram-negative anaerobic bacteria such as *Fusobacterium*, *Prevotella* and *Porphyromonas* predominated (Figure 4). These findings are consistent with three studies of mature plaque.^6,19,29^ For example, Ritz *et al.*^29^ investigated the temporal changes in the microbial composition of supragingival biofilms cultured for 9 days and found that the aerobic and facultative anaerobic bacteria *Streptococcus* and *Neisseria* predominated, whereas the proportion of *Fusobacterium* increased after 9 days. Moreover, recent molecular investigations of oral microbiota demonstrate variations in bacterial microbiota during the maturation of dental biofilms,^5,30,31^ consistent with our present results. Moreover, the shift in bacterial microbiota from aerobic to anaerobic conditions during biofilm formation may be attributed to the creation of niches that are beneficial for the proliferation and survival of obligate anaerobic bacteria.^18^

A study that investigated the effect of the time interval between brushing one’s teeth and the induction of gum inflammation found that brushing every 48 h increases plaque scores, although gingivitis does not develop.^32^ The authors speculated that this may be explained by the quantitative and qualitative changes in biofilms that occur approximately 48 h after initial growth. Our results agree with this finding. Thus, we show here that the proportion of anaerobic bacteria that cause gingivitis, such as *Fusobacterium, Prevotella* and *Porphyromonas,*^31,33^ increased after 48 h, indicating a shift to an environment that aggravates gingivitis. Thus, owing to the temporal variations of oral microbiota, we recommend that teeth should be brushed at least once every 48 h to prevent exacerbating gingivitis.

In conclusion, we used an *in situ* model of biofilm formation and detected a biphasic change in the phyla of biofilm-forming bacteria in the oral cavity of healthy subjects. Further, the bacterial population converted from facultative anaerobes to gram-negative anaerobes. It will be useful to compare the results of the present study with time-dependent changes in samples collected from individuals suffering caries or periodontal disease. Such studies will likely contribute to a comprehensive understanding of the bacterial microbiota involved in the health of the human oral cavity.

## Materials and methods

### Study subjects

The study included 10 healthy volunteers (three men and seven women) aged 26–30 years (mean 27.1±1.2 years) who were students of the Osaka University Graduate School of Dentistry during the study. We defined healthy volunteers as previously reported.^34^ Clinical or radiological signs of caries, gingivitis or periodontitis were not detected in any of the subjects. The volunteers abstained from antibiotics 6 months before the study commenced, and volunteers signed an informed consent form. The Ethics Committee of the Osaka University Graduate School of Dentistry approved the study design (H24-E4).

### *In situ* model of biofilms

**Figure 5:**
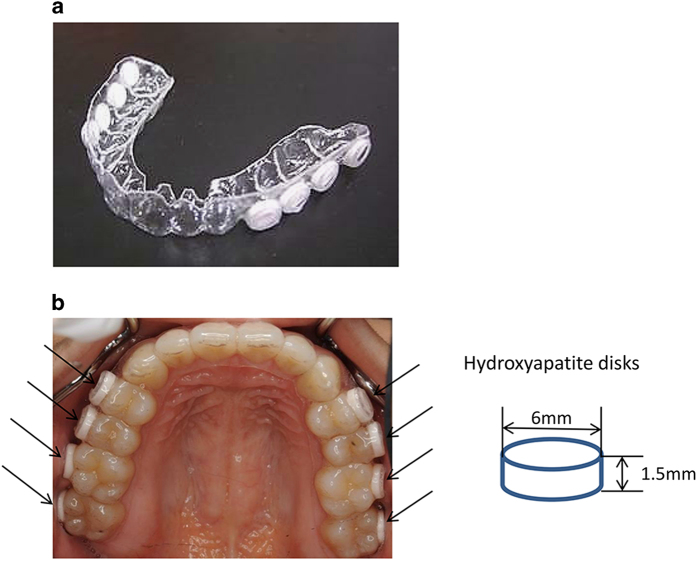
Image of upper-jaw acrylic appliance with eight hydroxyapatite (HA) disks. The diameter of the disks was 6 mm. The disks were fixed in the region of the premolars and molars toward the natural teeth on the buccal sides (arrows). (**a**) Appliance. (**b**) Appliance worn by a subject.

All volunteers wore a custom acrylic splint in their upper jaw for 96 h to form mature dental biofilms. In the splint, eight HA disks (6 mm diameter, 1.5 mm height) were inserted on the buccal side so that the biofilms were not peeled off by the tongue or cheek (Figure 5). The volunteers wore this splint for 96 h, except during meals and while brushing their teeth as previously described.^35^ The appliance was stored in a humidified chamber. After dental biofilms were formed, the disks were extracted without disrupting the adherent biofilm.

### Experimental protocol

**Figure 6:**
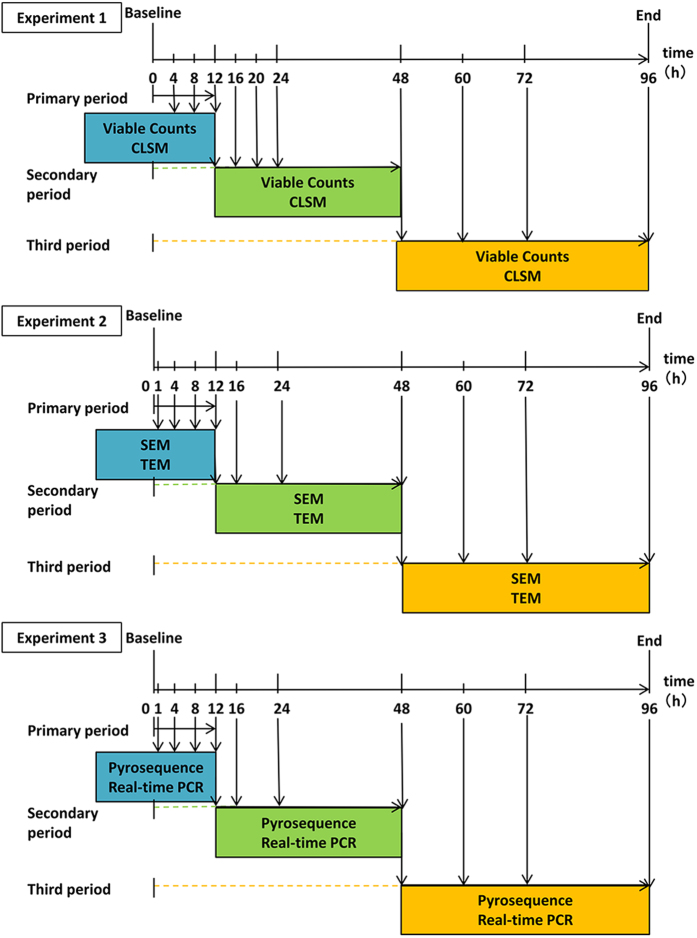
Experimental protocol. Experiment 1: determination of viable counts and three-dimensional analysis. Experiment 2: SEM and TEM observations. Experiment 3: DNA sequencing and real-time PCR quantification of biofilm-forming cells. CLSM, confocal laser scanning microscope; SEM, scanning electron microscope; TEM, transmission electron microscope; Viable counts, determination of viable count.

Dental biofilms formed on the HA disks were evaluated at 1, 4, 8, 12, 16, 24, 48, 60, 72 and 96 h. Because only eight disks fit into the appliance, we pooled our samples into three periods as follows: primary period (0–12 h), secondary period (12–48 h) and third period (48–96 h; Figure 6). At 1, 4, 8, 12, 16, 24, 48, 60, 72 and 96 h, two disks were extracted and used for two assays, for example, one disk was used to determine the number of viable bacteria and the other was used for confocal laser scanning microscope in Experiment 1.

### Determination of viable count

The samples were immersed in sterile distilled water, sonicated for 5 min and vortexed for 30 s. The specimens were used to inoculate Colombia blood agar (Becton, Dickinson and Company, Fukushima, Japan) and incubated aerobically or anaerobically for 48 h. Determination of viable counts was conducted by counting colonies. Each sample was tested in triplicate.

### Microscopy

Biofilm samples stained with the LIVE/DEAD BacLight Bacterial Viability Kit (Invitrogen, Carlsbad, CA, USA) were observed using a confocal laser scanning microscope (LSM700; Carl Zeiss, Oberkochem, Germany), as previously described.^36^ After reconstruction of confocal laser scanning microscope images using Imaris imaging software (Imaris 5.0.1; Bitplane AG, Zurich, Switzerland), determination of the thickness and volumes of live cells and dead cells was performed.

### SEM observations

The disks were prepared according to a previous protocol.^36^ The specimens were immersed in 50% Karnovsky’s solution (2.5% glutaraldehyde, 2% paraformaldehyde) for 30 min. They were then sequentially dehydrated in ethanol concentrations of 50, 70, 80, 90, 95 and 100% and freeze-dried. After sputter-coating with platinum, the samples were observed using an scanning electron microscope (JSM-6390LV; JEOL, Tokyo, Japan).

### TEM observations

Ultrastructural observations were performed as previously described.^37^ Briefly, the biofilms were fixed with 2.5% glutaraldehyde and 2% osmic acid, and dehydrated in a graded series of aqueous ethanol solutions followed by embedding in epoxy resin (Epon 812; NissinEM, Tokyo, Japan). Ultrathin sections were prepared and stained with 2% uranyl acetate and 0.4% Sato’s lead stain. Images were acquired using a transmission electron microscope (H-800; Hitachi, Tokyo, Japan).

### DNA sequencing

Two disks were collected at each time, and DNA was extracted using a PowerSoil DNA Isolation Kit (MO Bio Laboratories, Carlsbad, CA, USA). The V5–V6 region of the 16S rRNA gene was amplified using the primer set 784F: 5′- AGGATTAGATACCCTGGTA-3′ and 1061R: 5′- CRRCACGAGCTGACGAC-3′.^38^ Each library was prepared using an Ion Fragment Library Kit (Life Technologies, Grand Island, NY, USA) according to the manufacturer’s instructions. Sequencing was performed using an Ion PGM Sequencing 400 Kit (Life Technologies) with the Ion PGM sequencer (Life Technologies). The resulting sequences were analysed using the QIIME pipeline. The processed sequences were then clustered into operational taxonomic units defined according to a similarity cutoff=97% (see Supplementary Table S1) using UCLUST version 1.2.22q. Representative sequences for each operational taxonomic unit were classified taxonomically using RDP Classifier version 2.2 with the Greengenes Database.^39^

### Quantification of biofilm-forming cells

We used real-time PCR to determine the number of the biofilm-forming cells according to a published method.^40^ Briefly, the assays were performed in a total volume of 20 μl containing 10 μl of SYBR Select Master Mix (Applied Biosystems, Carlsbad, CA, USA), 0.5 μl each of the forward and reverse 16S V5–V6 primer set universal primers (final concentration, 900 nM each) and 1 μl of DNA. The Applied Biosystems 7500 Fast real-time PCR system (Life Technologies) was used, and standard curves were analysed using the universal primer set to amplify a serial dilution of *Streptococcus mutans* NCTC10449 plasmid DNA. The experiments were repeated thrice for each sample. The data were analysed using the 7500 System SDS Software Version 2.0.2 (Applied Biosystems).

### Statistical analysis

Differences in the number of viable cells, the thickness and the volumes of live and dead cells at each time point were evaluated using one-way analysis of variance and the Tukey–Kramer test implemented in JMP software (version10.0.2 2012 SAS Institute Inc., Cary, NC, USA). To perform statistical analysis of the temporal change of each genus, we used IBM SPSS Statistics (version 22.0, IBM SPSS Inc., Chicago, IL, USA) and the data are displayed as box plots. The differences between the populations of each genus at each time point were analysed using the Kruskal–Wallis and Steel–Dwass tests implemented in JMP software. Differences in the numbers of the biofilm-forming cells were analysed using one-way analysis of variance, Tukey–Kramer test. *P*<0.05 was considered significant.
