# Supplementary material for: Temporal dynamics of bacterial microbiota in the human oral cavity determined using an in situ model of dental biofilms
Source: NPJ Biofilms Microbiomes. 2016 Aug 10;2:16018–. doi: 10.1038/npjbiofilms.2016.18 (PMC5515266; doi:10.1038/npjbiofilms.2016.18)

Supplementary Online Text

Table S1. Good’s coverage value (%) and number of OTUs in each sample.

For each sample between 170 and 560 species-level OTUs (97% identity cutoff) were detected.

ID		1	2	3	4	5	6	7	8	9	10
**1h**	**Good's coverage value**	98.4%	98.8%	98.6%	98.2%	98.1%	98.4%	98.6%	98.3%	98.1%	97.9%
	**Number of OTUs**	340	318	347	402	480	388	383	387	458	560
**4h**	**Good's coverage value**	98.4%	98.5%	98.3%	98.1%	98.3%	98.7%	98.8%	98.8%	98.2%	98.6%
	**Number of OTUs**	377	338	361	424	334	288	274	252	345	313
**8h**	**Good's coverage value**	98.5%	98.9%	98.2%	98.7%	98.7%	98.6%	98.9%	99.2%	98.4%	98.6%
	**Number of OTUs**	337	241	345	284	275	284	257	170	335	314
**12h**	**Good's coverage value**	99.1%	98.7%	98.9%	98.6%	98.6%	98.4%	98.5%	98.6%	98.8%	98.7%
	**Number of OTUs**	185	254	280	279	315	311	295	312	235	303
**16h**	**Good's**	98.9%	98.5%	98.4%	98.6%	98.5%	98.4%	98.7%	99.1%	98.6%	99.0%

	**coverage value**										
	**Number of OTUs**	230	345	311	286	341	334	251	198	281	236
**24h**	**Good's coverage value**	98.7%	98.6%	98.6%	98.8%	98.6%	98.3%	99.0%	98.6%	98.7%	98.2%
	**Number of OTUs**	294	266	355	260	322	349	219	309	327	394
**48h**	**Good's coverage value**	98.5%	98.5%	99.1%	98.7%	98.7%	98.3%	98.9%	98.5%	98.7%	98.5%
	**Number of OTUs**	391	346	225	307	292	388	288	306	289	385
**60h**	**Good's coverage value**	98.5%	98.8%	98.5%	98.7%	98.5%	97.7%	99.1%	98.5%	98.7%	98.4%
	**Number of OTUs**	327	261	350	310	327	474	190	365	285	406
**72h**	**Good's coverage value**	98.6%	98.8%	98.6%	98.6%	98.5%	98.3%	98.8%	98.9%	98.9%	98.7%
	**Number of OTUs**	324	262	296	395	371	352	308	225	230	339
**96h**	**Good's coverage value**	98.6%	99.1%	98.8%	98.7%	98.5%	97.8%	98.7%	98.7%	98.6%	98.5%
	**Number of OTUs**	346	233	295	334	327	502	290	291	304	393

Table S2. Phylogenetic classification of OTUs.

The percentages indicate the average fraction of each genus in all healthy subjects at each time point. Only those genera above 1% of relative abundance are shown; together, they accounted for at least 85.2% of all differentially distributed OTUs.

genus	**1h**	**4h**	**8h**	**12h**	**16h**	**24h**	**48h**	**60h**	**72h**	**96h**
Actinomyces	3.80%	1.91%	0.84%	0.79%	0.77%	0.73%	0.75%	0.59%	0.54%	0.74%
Rothia	8.36%	13.8%	12.8%	13.3%	5.25%	7.04%	3.56%	3.53%	3.06%	1.95%
Capnocytophaga	2.31%	0.56%	0.43%	0.74%	1.59%	1.55%	3.53%	2.47%	4.94%	4.88%
Gemella	3.07%	2.72%	2.06%	1.64%	1.59%	2.70%	2.05%	1.57%	1.63%	2.00%
Streptococcus	28.9%	29.4%	31.8%	33.1%	32.4%	25.1%	15.8%	15.8%	15.8%	10.2%
Neisseria	7.09%	20.4%	21.0%	17.7%	26.9%	19.9%	18.7%	19.5%	25.1%	19.3%
Pasteurellaceae; Other	1.39%	2.09%	1.75%	1.51%	4.15%	8.08%	4.39%	5.29%	5.10%	5.19%
Haemophilus	4.67%	9.96%	10.9%	8.33%	7.81%	8.80%	6.91%	7.79%	10.0%	5.88%
Derxia	7.29%	3.12%	3.62%	4.45%	7.57%	1.83%	1.44%	0.80%	1.23%	1.64%
Porphyromonas	3.25%	2.83%	4.71%	8.15%	2.81%	10.7%	15.8%	15.8%	10.4%	11.7%
Prevotella	6.80%	3.30%	1.89%	2.01%	1.87%	4.73%	13.8%	10.3%	7.53%	12.4%
Fusobacterium	8.20%	3.47%	2.55%	2.76%	2.03%	2.89%	7.43%	9.44%	8.36%	15.0%

Figure S1 Relative abundance of bacterial taxa of all participants.

A) Relative abundance of bacterial phyla in each participant. The temporal changes in the relative amounts of each phylum are indicated. In all samples, the five most abundant phyla were detected. (n=10) B) Proportional abundance of bacterial genera in each participant. The temporal changes in the relative amounts of each genus are indicated. The most frequently detected taxa (above 1% of relative abundance) in each level are shown.

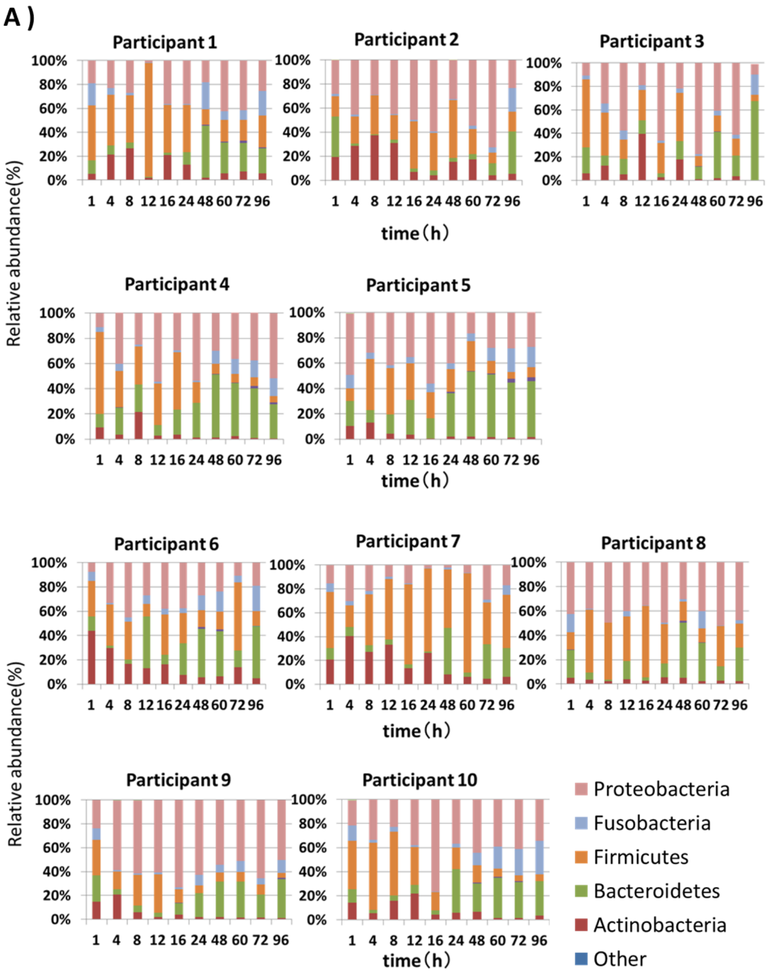

B)

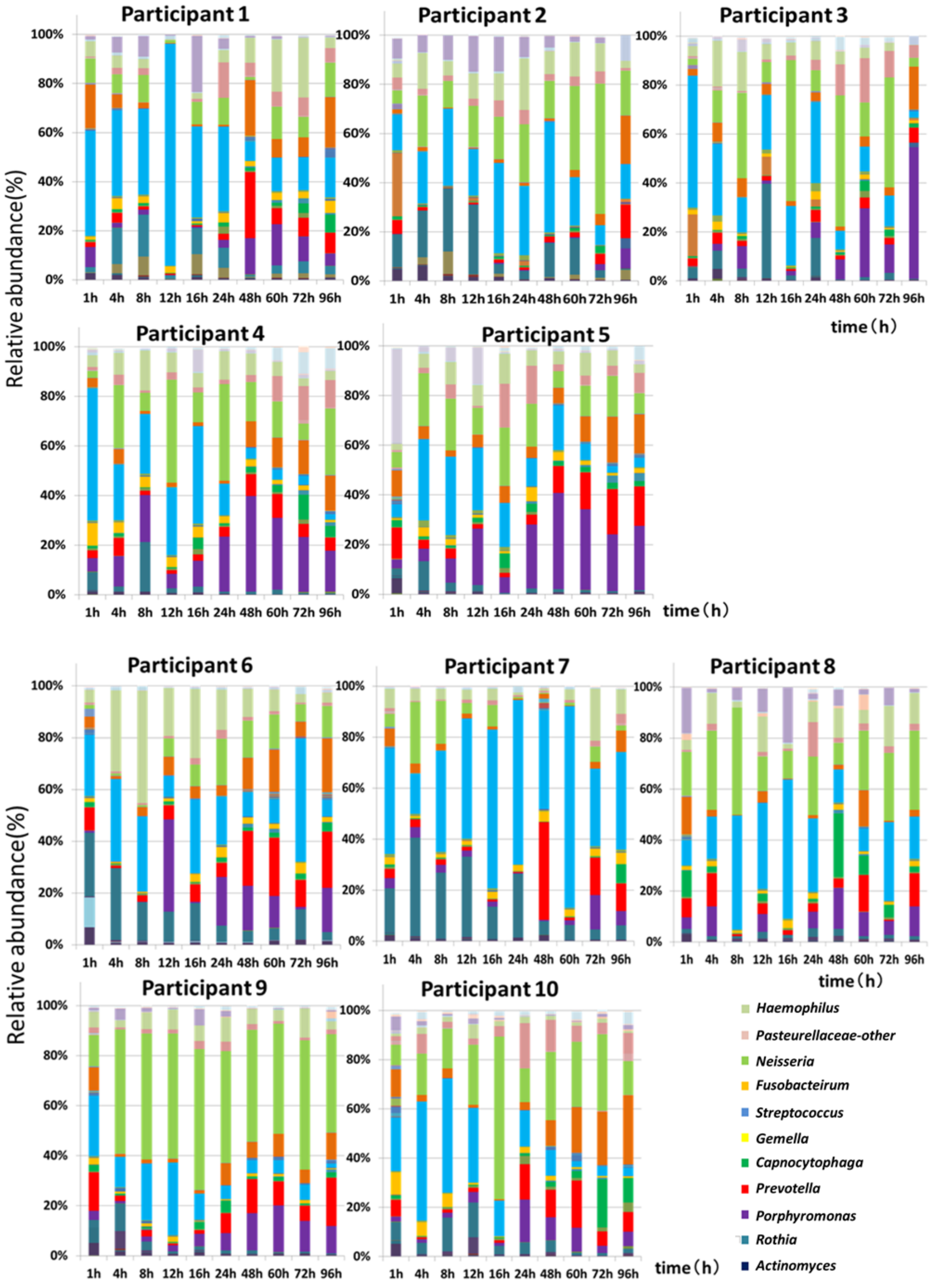

Participant 1
Participant 2
Participant 3
Participant 4
Participant 5
Participant 6
Participant 7
Participant 8
Participant 9
Participant 10
Relative abundance(%)
100%
80%
60%
40%
20%
0%
1h 4h 8h 12h 16h 24h 48h 60h 72h 96h
time（h）
Haemophilus
Pasteurellaceae-other
Neisseria
Fusobacteirum
Streptococcus
Gemella
Capnocytophaga
Prevotella
Porphyromonas
Rothia
Actinomyces

Figure S2 Temporal changes in diversity of each participant.

The boxplots indicate the Shannon diversity index in all subjects. The central line indicates the median. Error bars indicate the 10th and 90th percentiles. Circles represent outliers. a, b; They are those with a significant difference between opposite signs (*P* < 0.05).

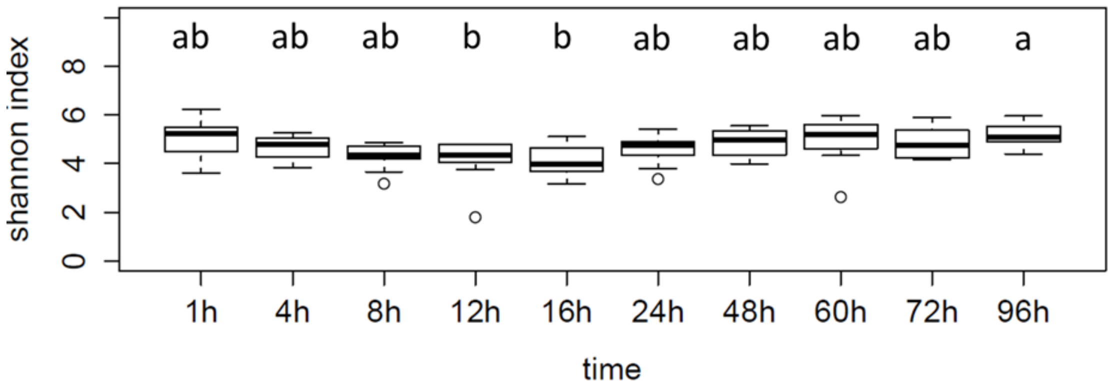

Supplement: Supplementary Information [file npjbiofilms201618-s1.pdf]
